# First detection and genomic analysis of *mcr-1*-positive *Salmonella* Infantis isolated from a broiler production system in the United Arab Emirates

**DOI:** 10.3389/fvets.2025.1592955

**Published:** 2025-08-26

**Authors:** Ihab Habib, Mohamed-Yousif Ibrahim Mohamed, Glindya Bhagya Lakshmi, Hassan Mohamed Al Marzooqi, Hanan Sobhy Afifi, Mohamed Gamal Shehata, Mohammed Elbediwi

**Affiliations:** ^1^Veterinary Public Health Research Laboratory, Department of Veterinary Medicine, College of Agriculture and Veterinary Medicine, United Arab Emirates University, Al Ain, United Arab Emirates; ^2^Food Research Section, Applied Research and Capacity Building Division, Abu Dhabi Agriculture and Food Safety Authority (ADAFSA), Abu Dhabi, United Arab Emirates; ^3^Department of Food Technology, Arid Lands Cultivation Research Institute (ALCRI), City of Scientific Research and Technological Applications (SRTACITY), Alexandria, Egypt; ^4^Department of Evolutionary Biology, Institute for Biology, Freie Universität Berlin, Berlin, Germany; ^5^Animal Health Research Institute, Agriculture Research Centre, Cairo, Egypt

**Keywords:** United Arab Emirates, antibiotic resistance, *Salmonella*, IncX4, Colistin

## Abstract

This study reports the first detection of *mcr-1.1*-mediated colistin resistance in *Salmonella enterica* serovar Infantis from a commercial broiler farm in the United Arab Emirates (UAE). Two *S. infantis* isolates (SAL_93 and SAL_94) were recovered from caecal droppings and characterized using whole-genome sequencing (WGS). Genomic analysis revealed a single-nucleotide polymorphism (SNP) difference between them, confirming their close epidemiological relationship. Both isolates belonged to multilocus sequence type 32 and exhibited multidrug resistance (MDR), including resistance to colistin (MIC = 4 mg/L) and ciprofloxacin (MIC = 0.5 mg/L). Notably, the *mcr-1.1* gene was detected on a conjugative IncX4 plasmid. Additionally, the isolates harbored a large (275,043 bp) conjugative IncFIB plasmid carrying multiple AMR genes, including *aadA1*, *sul1*, *tet(A)*, *qacEdelta1*. Bioinformatic analysis showed a high identity for globally reported *mcr-1.1*-carrying IncX4 plasmids. The investigation of virulence-associated factors in the studied isolates identified 162 potential virulence-related genes. These included genes linked to the type 3 secretion system, specifically those encoded by pathogenicity island-1 (SPI-1). However, multiple genes linked to the second type 3 secretion system, encoded by SPI-2, were absent in all isolates. These findings suggest a potential risk of horizontal gene transfer in poultry production. Given these risks, the UAE’s recent ban on colistin in veterinary medicine marks a crucial step in mitigating AMR transmission within a One Health framework.

## 1 Introduction

The emergence of colistin resistance has become a global concern, affecting both human health and food-producing animals. In human health, colistin has been recommended as a last-line defense against infections by multidrug-resistant (MDR) pathogens (1). Within animal health, colistin has long served as a therapeutic and prophylactic antimicrobial against infections caused by Gram-negative bacteria, such as *Salmonella* spp., which are prevalent in both human and animal health sectors (2). Among the various *Salmonella* serovars, *Salmonella enterica* serovar Infantis ranks among the leading causes of human infections worldwide (3). The increasing frequency of *S. infantis* illnesses in some regions is further complicated by the proliferation of MDR strains, including those capable of producing *β*-lactamases, as seen in the recent dissemination of *S. infantis* serovar in broiler farms, poultry meat, and human gastroenteritis cases (4).

In addition to its impact on animal health, *S. infantis* poses a significant zoonotic risk, with multiple outbreaks linked to contaminated poultry products and subsequent human infections reported globally (e.g., Europe, North America, and Asia) (3–5). The serovar’s ability to harbor resistance and virulence determinants makes it a priority pathogen in integrated food safety monitoring programs. Despite the growing poultry industry in the United Arab Emirates (UAE), data on the epidemiology of *S. infantis* in broiler production and its potential role in zoonotic transmission remain scarce, underscoring a critical research gap.

Since the identification of the gene *mcr-1* in China in 2015, this plasmid-mediated colistin resistance determinant has been linked to the rising prevalence of colistin resistance in food, livestock, and people internationally (1). In the UAE, previous studies have documented the presence of the gene *mcr-1* in colistin-resistant *E. coli* and *S.* Minnesota strains isolated from supermarket poultry (6, 7). This study presents the first documented occurrence of *mcr-1*-mediated colistin non-susceptibility in an MDR *S. infantis* isolate, which also exhibits resistance to broad-spectrum cephalosporins. To our knowledge, this is the first report of *mcr-1*-positive MDR *S. infantis* in UAE broiler farms, highlighting the urgent need to monitor emergent resistance in local production systems and its potential implications for food safety and public health. The resistant strains were recovered from one broiler farm as part of a baseline microbiological assessment of the broiler supply chain in Abu Dhabi.

## 2 Materials and methods

### 2.1 Study setting

The study site was a standard medium-sized broiler farm in the UAE, housing approximately 20,000 Lohmann broilers in a closed, climate-controlled system. The farm followed standard all-in/all-out flock management, automated feeding and watering systems, and strict biosecurity measures with a contracted (not resident) routine veterinary oversight. The birds were reared on deep litter flooring composed of wood shavings, with a stocking density of approximately 33 kg/m^2^. The farm operated on a 30 to 35-day production cycle; such a short production cycle is a typical practice in the UAE broiler farming sector in response to consumer preference of small-sized (net weight of ≤1,000 gm). For sample collection, caecal droppings were systematically collected from various locations within the broiler house to ensure a representative sampling of the flock’s gut microbiota. Sampling sites were selected to cover different zones, including areas near feeders and water lines, corners of the house, and the center where birds tend to aggregate. Fresh caecal droppings, distinguishable by their typical dark brown to mahogany color, pasty, and more homogenous texture than the firmer, segmented regular feces, were identified and collected using sterile scoops. A pooled sample of a total of 50 caecal droppings was collected to reach a target quantity of approximately 150 g (or about the size of a tennis ball, ~8 cm diameter), as typically applied in national and regional monitoring programs for Salmonella detection in poultry (8). The samples were immediately placed into a sterile capped container, transported in a cold chain (4°C), and processed within four to six hours of collection for microbiological and genomic analysis.

### 2.2 Microbiological isolation and characterization

The container with collected caecal droppings was weighed, and an equal portion of buffered peptone water (BPW) (Oxoid, Basingstoke, Hampshire, UK) was added to attain a 1:1 diluent-to-sample ratio. The mixture was homogenized for one minute using a BagMixer (Interscience, St Nom, France). Then, 50 mL of this homogenized sample was combined with 200 mL of BPW and homogenized again, resulting in a final dilution factor of 1:10 (w/v). From this suspension, 25 mL was directly incubated at 37°C for 18 h for pre-enrichment. No plating of serial dilutions was performed at this stage, as the method relies on selective enrichment and subsequent isolation on selective media following pre-enrichment, in accordance with *Salmonella* detection protocol of the ISO 6579-1 (2017) (9). For pre-enrichment, 100 μL was sub-cultured in modified semi-solid Rappaport Vassiliadis (MSRV) medium (Oxoid, UK) and incubated at 41.5°C for 48 h. MSRV plates were checked at 24 h for a migration zone (turbid, white halo >10 mm) and rechecked at 48 h if absent. Following pre-enrichment, streaking was performed on xylose lysine deoxycholate agar (Oxoid, UK) with incubation at 37°C for 24 h (10). Up to five suspected colonies from selective media were transferred to nutrient agar (Oxoid, UK) and incubated at 37°C for 24 h. Purified colonies were identified at the species level using MALDI-TOF MS with the Autobio-MS-1000 (Autobio Diagnostics, China).

The characterization of antimicrobial resistance using minimum inhibitory concentrations (MICs) technique was carried out utilizing Sensititre™ EUVSEC3 plates (Thermofisher Scientific, UK), following the protocol provided by the manufacturer. The assessment of resistance was based on epidemiological cut-off values (ECOFFs) stipulated by the European Committee on Antimicrobial Susceptibility Testing (EUCAST) guidelines (11).

### 2.3 Whole-genome sequencing (WGS)

Genomic DNA was prepared using the Wizard® DNA Kit (Promega, USA), followed by a quality assessment (12). Short-read sequencing was outsourced to Novogene (UK) and conducted on the NovaSeq platform using 2 × 150 bp paired-end reads. Library preparation followed standard Illumina protocols. Sequencing generated assemblies with an N50 of 203,983 bp, genome sizes of ~5.00 Mbp, and 52–56 contigs per genome (Table 1), consistent with high-quality *Salmonella* WGS data reported in previous studies (3, 12). Genome analysis was performed with the cloud-based Solu platform v1.0.229 (accessed 15 February 2025) (13), which integrates a suite of validated pipelines for bacterial genomics. Species confirmation was achieved with Kraken2 v2.1.2 followed by Bracken re-estimation, serovar prediction employed the SISTR v1.1.1 workflow, and multilocus sequence types were assigned with mlst v2.23.0 using the *Salmonella* PubMLST scheme (13). Antimicrobial-resistance genes and point mutations were detected in parallel with AMRFinderPlus v3.11, plasmid replicons were identified with PlasmidFinder v2.1, and virulence loci were screened using VirulenceFinder. Default parameters were applied throughout, ensuring reproducible and transparent outputs for each analytical step (13). All raw sequencing data have been deposited in the National Center for Biotechnology Information (NCBI) under BioProject number PRJNA1231376.

**Table 1:** Whole-genome sequencing assembly details and antimicrobial resistance genes and MOB-suite based predicted plasmid characters.

Whole-genome sequencing assembly quality metrics								
Isolate	Serovar	Sequence type	Genome size	Number of contigs	GC%	N50	Genome fraction	SNP distance
SAL_93	Infantis	ST-32	5.00 Mbp	56	52.05	203,983	88.94%	Reference
SAL_94	Infantis	ST-32	5.00 Mbp	52	52.05	203,983	88.93%	1
Antimicrobial resistance genes and in silico plasmid characterization								
MOB Cluster	Size (bp)	AMR genes		Replicon types	Relaxase types	mpf	oriT	Mobility
		Number	Identity					
PlasmidAC358	275,043	4	*aadA1* *qacEdelta1* *sul1* *tet(A)*	IncFIB	MOBP	MPF_I	MOBP	conjugative
PlasmidAA619	32,969	1	*mcr-1.1*	IncX4	MOBP	MPF_T	-	conjugative
PlasmidAC044	9,405	2	*bla*_TEM-1_ *erm(B)*	-	-	-	-	non-mobilizable

### 2.4 *In silico* plasmid characterization and mobility prediction

Plasmids carrying *mcr-1* were analyzed by extracting plasmid-associated contigs from genome sequence data using Plasmid SPAdes with default settings (14). The reconstructed contigs were then compared against the non-redundant NCBI database to identify the closest matching plasmid, ensuring 99–100% coverage and 100% sequence identity (12).

Plasmid mobility was analyzed using the MOB-suite platform (v3.1.9). Identified plasmid scaffolds from WGS data were matched against the MOB-clusters database to determine their closest counterparts (15). Plasmids were classified into MOB-clusters and categorized as “Conjugative,” “Mobilizable,” or “Non-mobilizable” based on their mobility potential. Additionally, relaxase gene clusters were identified, as they play a key role in plasmid transfer by recognizing and cleaving the origin of transfer (oriT) sites, enabling the horizontal spread of antimicrobial resistance genes (15).

## 3 Results

### 3.1 Characteristics of *mcr-1*- harboring *Salmonella* Infantis isolates

Two *Salmonella enterica* serovar infantis strains (SAL_93 and SAL_94) were identified from a pooled sample of fresh caecal droppings collected from the environment of a commercial broiler farm in the UAE (Table 1). WGS confirmed their classification using a computational analysis performed using Solu platform v1.0.229 with standard settings (13). The genome-level relatedness of these strains was evident, with only a Single Nucleotide Polymorphism (SNP) difference between them (Table 1). As shown in Table 1, both strains belonged to multilocus sequence type 32.

The minimum inhibitory concentration (MIC, Sensititre™ EUVSEC3 panel) of colistin for these isolates was determined to be 4 mg/L. Additionally, both isolates exhibited MDR profile to a wide range of antimicrobials, including resistance to ampicillin (MIC = 32 mg/L), azithromycin (MIC = 32 mg/L), chloramphenicol (MIC = 32 mg/L), ciprofloxacin (MIC = 0.5 mg/L), nalidixic acid (MIC = 64 mg/L), sulfamethoxazole (MIC = 512 mg/L), and tetracycline (MIC = 32 mg/L).

### 3.2 In silico plasmids reconstruction and *mcr-1.1* gene context

As indicated in Table 1, the *mcr-1.1* gene identified in these isolates was found on a predicted conjugative plasmid classified as MOB-cluster AA619 (replicon type IncX4). These *S. infantis* isolates also carried a conjugative plasmid classified as MOB-cluster AC358 (replicon type IncFIB), which harbored multiple antimicrobial resistance (AMR) genes, including *aadA1* (conferring streptomycin resistance), *qacEdelta1* (associated with quaternary ammonium compound resistance), *sul1* (resistance to sulfonamides), and *tet(A)* (tetracycline resistance). The former predicted plasmid is notably large, measuring 275,043 base pairs. Additionally, a chromosomal mutation (*gyrA_S83Y*) was identified in the gyrase gene of the two strains. These strains also carried *mdsA* and *mdsB*, which encode the MdsABC efflux system. Moreover, the *β*-lactamase gene *bla*_TEM-1_ and the *erm(B)* gene were identified and predicted to be on a non-mobilizable plasmid (Table 1).

### 3.3 Comparison of *mcr-1.1* gene–environment with internationally related plasmids

**Figure 1:**
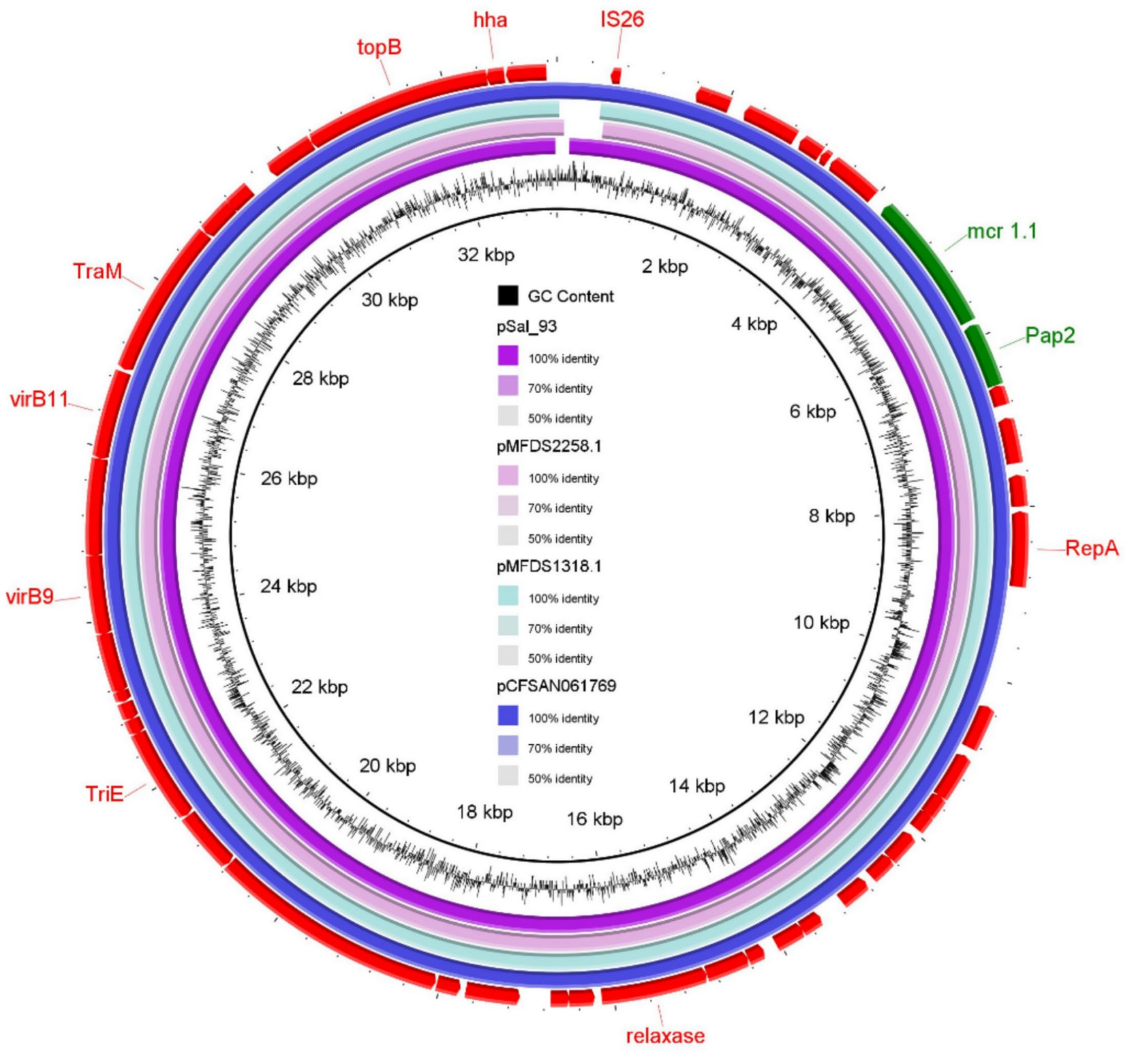
Genomic comparison of *mcr-1.1* gene–environment between *Salmonella* Infantis strain SAL_93 isolated from a broiler farm in the United Arab Emirates and particular internationally concorded plasmids.

Plasmid contigs from these isolates exhibited a 100% identity match with previously documented IncX4 plasmids carrying *mcr-1.1*, including pCFSAN061769_01 (100% coverage, detected in *E. coli* from beef sausage in Egypt) and pMFDS1318.1 and pMFDS2258.1 (99% coverage, identified in *E. coli* from pig farms in Korea) (Figure 1). The genetic localization of the *mcr-1.1* gene showed that a *PAP-2* family protein-coding gene was positioned downstream of *mcr-1.1* in *S. infantis*, while the nearby transposable element was identified as IS-26 (Figure 1).

### 3.4 Putative virulence determinants

WGS uncovered 162 potential virulence-related genes in the *mcr-1.1*-bearing *S. infantis* strains (Sal_93 and Sal_94) (Table 2). These isolates lacked genes typically linked to capsule formation, invasion, immune evasion, antiphagocytic activity, autotransport, serum resistance, stress adaptation, or toxin production. However, they retained virulence factors associated with adhesion (fimbrial and nonfimbrial) and biofilm formation. The *csg* (curli-specific gene) operons involved in surface adherence, cell aggregation, environmental persistence, and biofilm development were present. Both isolates possessed virulence elements characteristic of *Salmonella* pathogenicity islands SPI-1 and SPI-2 but lacked the SPI-2-associated genes *ssaS* and *sseA* (Table 2).

**Table 2:** Putative virulence factors and their associated gene clusters presented in the *mcr-1.1* positive *Salmonella* Infantis strain SAL_93 isolated from a broiler farm in the United Arab Emirates and particular internationally concorded plasmids.

Virulence factor classes	Gene clusters present	Gene clusters absent
Capsule		Vi antigen
Fimbrial adherence	*Agf/Csg, Bcf, Fim (except fimW and fimY), Lpf, Stb, Saf, (except sfA and sfD), Stc (except stcA and stcD), Std, Stf, Sth, Sti, Tcf (except tdfA)*	*Pef, Peg, Sef, Sta, Ste, Stk*
Macrophage inducible genes	*migneg14*	*migneg5*
Magnesium uptake	*mgtB, mgtC*	
Nonfimbrial adherence	*misL, ratB, shdA, sinH*	
Regulation	*phoP, phoQ*	
Secretion system	TTSS (SPIneg1 encode): all genes presented	
	TTSS (SPIneg2 encode): missing *ssaS and sseA*	
	TTSS effectors translocated via both systems: missing *sspH1*	
	TTSSneg1 translocated effectors: missing *sopE*	
	TTSSneg2 translocated effectors: missing *gogB, sopD2, spiC/ssaB, spvC, spvD, and ssel/srfH*	
Serum resistance		*Rck*
Stress adaptation		*SodCI*
Toxin		*SpvB*, and Typhoid toxin
Adherence	K88 fimbriae (Escherichia)	
	Type IV pili (Yersinia)	
Iron uptake	Yersiniabactin siderophore (Escherichia)	Aerobactin siderophore (Escherichia)
	Yersiniabactin (Klebsiella)	Iron/manganese transport (Escherichia)
	Yersiniabactin (Yersinia)	Salmochelin siderophore (Escherichia)
		Salmochelin synthesis and transport (Shigella)
Invasion		*ibeB*
Immune evasion		*gtrA*
Antiphagocytosis		*probable wbaZ*
Autotransporter		*ehaB*

## 4 Discussion

This study reports for the first time in the UAE the detection of colistin-resistant *S. infantis* strains harboring the *mcr-1.1* gene recovered from broiler farms. Previously, colistin resistance linked to *mcr* genes in UAE poultry had only been associated with *Salmonella* Minnesota isolates from farms and retail meat products (6). The *mcr-1.1* gene context here was located on an IncX4 conjugative plasmid, well-documented for its capability to facilitate horizontal transfer of *mcr*-mediated colistin resistance across *Enterobacteriaceae* and *Salmonella* serovars (6, 16). Additionally, the isolates contained an exceptionally large IncFIB plasmid, aligning closely with pESI-like plasmids, known for enhancing the adaptability and survival of *S. infantis* in various environments (17).

*S. infantis* identified in this study belonged to the dominant sequence type commonly reported before as the most prevalent infantis subtype in UAE retail chicken products (3). In Europe, *S. infantis* has been recognized as an emerging serovar., frequently found in poultry production systems, ranking just behind *S. enteritidis* and *S. typhimurium* in prevalence (5, 17). The antimicrobial resistance profile of these isolates mirrors earlier observations from the UAE poultry sector, where over 95% of *S. infantis* isolates exhibited multidrug resistance (MDR) (3). While colistin resistance had not previously been identified in *S. infantis* in the UAE, its detection in this study raises significant public health concerns, as it potentially restricts available therapeutic options for treating severe human infections, especially those involving MDR strains transmitted through the food chain (18).

The genetic context of the *mcr-1.1* gene was notably close to the transposable element IS-26, a mobile genetic element well-known for facilitating genetic transposition through replicative or conservative mechanisms, playing a crucial role in the spread of antimicrobial resistance (AMR) genes (19). This genetic context, coupled with the presence of conjugative plasmids harboring multiple AMR genes, highlights the potential for further dissemination and challenges efforts aimed at controlling MDR bacterial pathogens, including *S. infantis*.

The isolates resisted critically important antibiotics, such as colistin, ciprofloxacin, and azithromycin, underlining the potential implications for human health. Furthermore, the detection of genes promoting the MdsABC efflux system (*mdsA* and *mdsB*), members of the resistance-nodulation-cell division (RND) superfamily, further emphasizes the robustness of resistance mechanisms in these strains (20). Identifying conjugative plasmids carrying such AMR genes in *mcr*-positive *S. infantis* from UAE broiler farm environment raises concerns regarding potential cross-transmission between poultry and humans and underscores the role poultry-associated strains may play in facilitating horizontal gene transfer (21).

Despite the absence of traditional virulence determinants associated with immune evasion, toxin production, and serum resistance, the isolates retained virulence factors related to adhesion and biofilm formation, notably the *csg* operons (22). The presence of virulence genes characteristic of *Salmonella* pathogenicity islands SPI-1 and SPI-2 (excluding specific SPI-2-associated genes *ssaS* and *sseA*) suggests these strains maintain pathogenic potential primarily through biofilm-mediated environmental persistence and intracellular survival (23, 24). These findings indicate that pathogenicity in these *S. infantis* isolates may rely more on colonization efficiency and persistence rather than conventional virulence pathways.

## 5 Conclusion

These findings highlight the importance of strengthening antimicrobial resistance surveillance through a One Health approach, integrating human, food, and environmental monitoring systems. In a proactive step toward addressing the growing threat of colistin resistance, the UAE Ministry of Climate Change and Environment implemented a complete ban on its use in veterinary medicine as of February 2024 (https://www.moccae.gov.ae/en/our-services/product-and-materials-page.aspx). By adopting this measure, the UAE contributes proactively to international efforts to combat antimicrobial resistance, potentially reducing the spread of resistant bacteria and preserving colistin as a vital last-resort antibiotic for human medicine.

## Data Availability

The data presented in the study are deposited in the National Center for Biotechnology Information (NCBI) under BioProject number PRJNA1231376 (https://www.ncbi.nlm.nih.gov/bioproject/?term=PRJNA1231376).
